# Enhancement in the Catalytic Activity of Pd/USY in the Heck Reaction Induced by H_2_ Bubbling

**DOI:** 10.3390/molecules16010038

**Published:** 2010-12-24

**Authors:** Kazu Okumura, Takuya Tomiyama, Sayaka Moriyama, Ayaka Nakamichi, Miki Niwa

**Affiliations:** Department of Chemistry and Biotechnology, Tottori University, 4-101, Koyama-cho Minami, Tottori, Japan

**Keywords:** Heck coupling reaction, USY zeolite, palladium, H_2_ bubbling, acid property

## Abstract

Pd was loaded on ultra stable Y (USY) zeolites prepared by steaming NH_4_-Y zeolite under different conditions. Heck reactions were carried out over the prepared Pd/USY. We found that H_2_ bubbling was effective in improving not only the catalytic activity of Pd/USY, but also that of other supported Pd catalysts and Pd(OAc)_2_. Moreover, the catalytic activity of Pd/USY could be optimized by choosing appropriate steaming conditions for the preparation of the USY zeolites; Pd loaded on USY prepared at 873 K with 100% H_2_O gave the highest activity (TOF = 61,000 h^−1^), which was higher than that of Pd loaded on other kinds of supports. The prepared Pd/USY catalysts were applicable to the Heck reactions using various kinds of substrates including bromo- and chloro-substituted aromatic and heteroaromatic compounds. Characterization of the acid properties of the USY zeolites revealed that the strong acid site (OH_strong_) generated as a result of steaming had a profound effect on the catalytic activity of Pd.

## 1. Introduction

Heck reactions have been recognized as an important, useful and versatile methodology for the synthesis of aryl alkenes via arylation or vinylation of olefins. The resulting reaction products are widely utilized for production of pharmaceuticals, organic electroluminescent devices, and liquid crystals [1], therefore much effort has been devoted to developing Pd catalysts active in the Heck reaction. Furthermore, the reaction proceeds under mild conditions to yield products with high efficiency. Numerous Pd complexes, including palladacycles [2,3] and *N*-heterocyclic carbenes [4], have been developed for use in these reactions. Supported Pd catalysts are other candidates for use in Heck reactions, which compared to the homogeneous ones, are easily prepared and readily separated from the products. For this purpose, Pd has been supported on various materials, including activated carbon [5,6], zeolites [7,8,9,10] and polymers such as polyethylene glycol [11,12]. Zeolites are expected to be an efficient support to accommodate active metal centers because they have large surface area and uniform micropores. Among the various types on zeolites, faujasite (FAU)-type ones are the most promising for use as a support for Pd because they have large supercages with diameters of *ca.* 1.3 nm, which can be regarded as nano-sized flasks. Indeed, we found that Pd loaded on H-Y type zeolites was suitable for use in Heck reactions [8]. Furthermore, USY zeolites exhibited excellent catalytic activity in a Suzuki-Miyaura reaction [13,14]. A remarkable improvement in the catalytic activity in a Suzuki-Miyaura reaction was achieved by continuously bubbling 6% H_2_ through the system during the reaction, probably due to the promotion of the reductive elimination step caused by dissolved H_2_. Pd K-edge extended X-ray absorption fine structure (EXAFS) analysis revealed the formation of atomic Pd species after H_2_ bubbling in *o*-xylene. In general, USY zeolites are prepared by steam treatment of Y-type (FAU structure) zeolites ion-exchanged with NH_4_^+^ cations (NH_4_-Y). Tuning of the acid properties of USY is also possible by changing the steam-treatment conditions, *i.e.*, temperature, time, and H_2_O vapor concentration. A further improvement in activity was achieved after optimization of the steaming conditions for USY [15]. Here, we tried to apply the Pd/USY catalysts in Heck reactions, focusing on the effects induced by continuous H_2_ bubbling during the reactions as well as the NH_4_-Y steaming conditions used to prepare the USY zeolite supports.

## 2. Results and Discussion

### 2.1. Structural Characterizations of USY Zeolites Prepared Under Different Conditions

**Figure 1:**
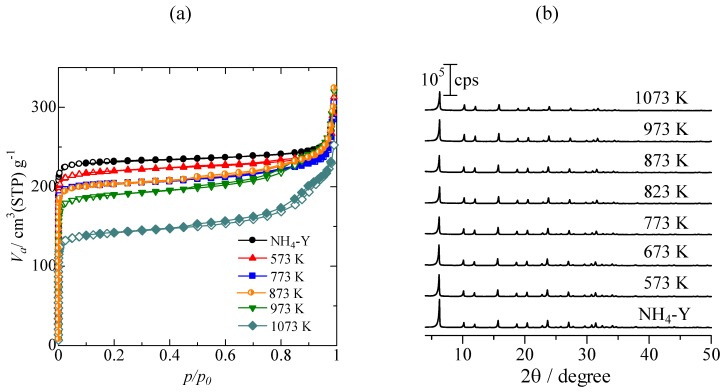
(a) N_2_ adsorption isotherms. Open symbols: adsorption branches, closed symbols: desorption branches; (b) XRD patterns of USY zeolites prepared by steaming of NH_4_-Y zeolites with 100% H_2_O at different temperatures.

It has been reported that steam treatment of NH_4_-Y under severe conditions results in the formation of mesopores as a result of dealumination of the Y-type zeolite framework [16]. Figure 1(a) shows N_2_ adsorption isotherms of NH_4_-Y and the USY prepared by steam treatment with NH_4_-Y with 100% H_2_O at different temperatures. Although a gradual decrease in N_2_ adsorption capacity was observed up to a steaming temperature of 1,073 K, mesopore formation was not obvious in every USY sample, except for the USY prepared at 1,073 K; thus the possibility of mesopores participating in the Pd catalytic reactions described in the following sections may be ruled out. The BET surface areas of NH_4_-Y and the USY prepared at 873 K were calculated to be 710 and 630 m^2^ g^−^^1^, respectively.

X-ray diffraction (XRD) patterns of NH_4_-Y and USY zeolites prepared by steaming of NH_4_-Y are presented in Figure 1(b). Diffraction patterns characteristic of FAU-type zeolite was seen in every sample. Although a slight decrease in the intensity of diffraction peaks was observed, it was confirmed that the FAU-type structure was preserved, even after the steaming with 100% H_2_O at 1,073 K.

### 2.2. Acid Properties of USY Zeolites Prepared under Different Conditions

**Figure 2:**
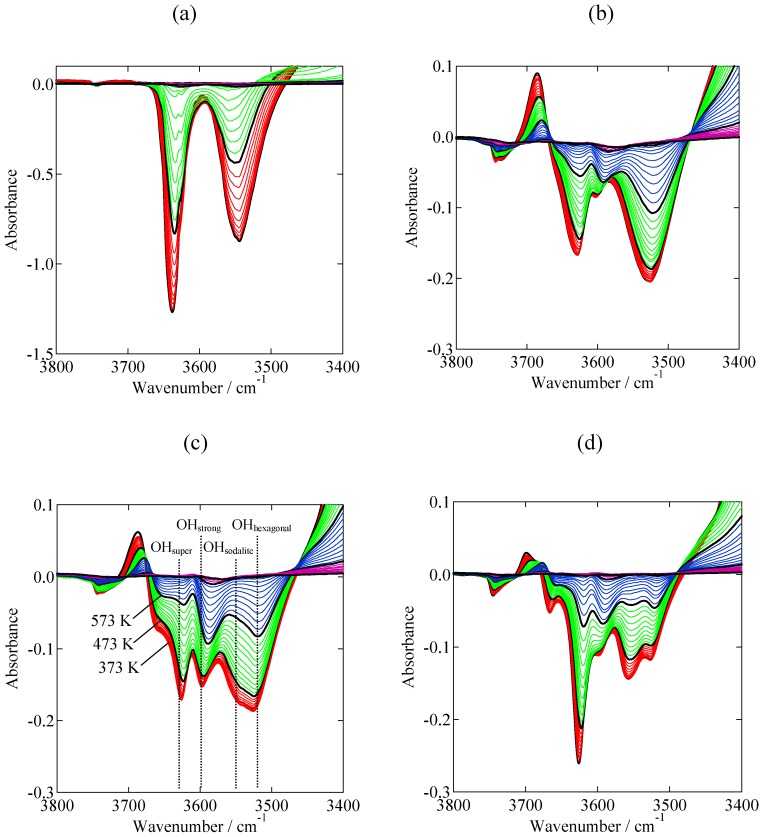
OH region of the difference IR spectra measured on (a) H-Y and (b-d) USY (prepared by steaming with 100% H_2_O for 1 h) with adsorbed ammonia during the elevation of temperature from 373 to 773 K. Spectra were taken every 10 K. Bold lines show the spectra measured at 373, 473, 573 K, 673 K and 773 K. Steaming conditions: (b) 773 K (c) 873 K (d) 973 K.

Figure 2 shows the difference IR-TPD spectra of H-Y and USY (steam-treatment conditions: 100% H_2_O at 773, 873, 973 K for 1 h) with adsorbed NH_3_, as the temperature was raised from 373 to 773 K. In these figures, the OH stretching region of the adsorbed NH_3_, and the subsequent TPD, is enlarged. The OH stretching region consisted of five kinds of OH group: OH groups in the supercage (3,630 cm^−1^), extra-framework Al species (3,609 cm^−1^), sodalite cage (3,550 cm^−1^), hexagonal prism (3,520 cm^−1^), and strong acid sides characteristic of USY (3,598 cm^−1^). In the spectra of USY zeolites, a new negative band was seen at 3,598 cm^−1^ (OH_strong_), which was not observed in the unmodified H-Y (Figure 2a). The intensity of the OH_strong_ band increased with increasing temperature, so it is assumed that this band was created as a result of the steam treatment. Table 1 and Table 2 list the amount and strength of the each acid site determined based on the IR spectra and MS that was simultaneously measured during TPD of ammonia, respectively. The detailed data analysis method was described elsewhere [15]. The amount of the OH_strong_ band was largest when the USY was prepared at 873 K. At this temperature, the resulting Pd/USY also exhibited the highest activity in Heck reactions, as will be mentioned later. Excessive steam treatment at 973 K resulted in a decrease in the number of strong acid sites. Therefore, it is assumed that the interaction with OH_strong_ band was responsible for the evolution of high activity of Pd. This tendency was quite different from that of other kinds of acid sites (OH_super_, OH_sodalite_, OH_hexagonal_); the amounts of these acid sites decreased with increased steaming temperature. It is worth to mention that the acid strength of OH_strong_ (153–159 kJmol^−^^1^) was higher than those of other OH sites (Table 2).

**Table 1:** Amount of Brønsted acid sites in USY zeolites determined by NH_3_ IRMS-TPD methods.

Sample	OH_super_	OH_strong_	OH_sodalite_	OH_hexagonal_
	/ mol kg^−1^	/ mol kg^−1^	/ mol kg^−1^	/ mol kg^−1^
H-Y	1.20	-	0.38	0.31
USY (773 K)	0.23	0.17	0.08	0.11
USY (873 K)	0.10	0.18	0.05	0.04
USY (973 K)	0.10	0.13	0.04	0.02

**Table 2:** Strength of Brønsted acid sites in USY zeolites determined by NH_3_ IRMS-TPD methods.

Sample	OH_super_	OH_strong_	OH_sodalite_	OH_hexagonal_
	/ kJ mol^−1^	/ kJ mol^−1^	/ kJ mol^−1^	/ kJ mol^−1^
H-Y	112	-	117	107
USY (773 K)	139	159	152	151
USY (873 K)	141	158	144	143
USY (973 K)	141	153	142	142

### 2.3. Pd K-edge EXAFS of Pd/USY Reduced with Bubbling H_2_ in DMAc

**Figure 3:**
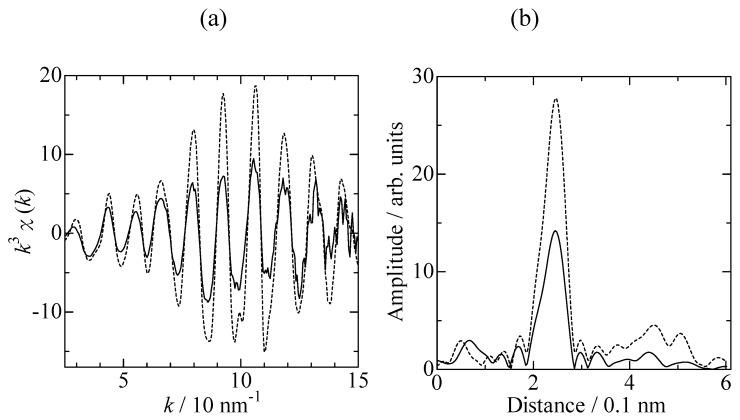
Pd-K edge EXAFS (a) *k^3^χ*(*k*) and (b) their Fourier transforms of Pd/USY reduced with bubbling 6%-H_2_ in DMAc (continuous line) and Pd foil (dotted line).

In order to obtain some insight into the structure of the active Pd species, Pd K-edge EXAFS data was collected under *in situ* conditions. Figure 3 shows Pd K-edge EXAFS spectra of Pd/USY (steaming temperature: 873 K) measured with 6%-H_2_ bubbling in dimethylacetamide (DMAc) and Pd foil. The Pd–Pd bond characteristic of metallic Pd appeared at 0.22 nm in the Fourier transform (phase shift uncorrected). Curve fitting analysis using Pd foil as the reference revealed that the coordination number (CN) of the metallic Pd was 6.5, implying the formation of Pd clusters. The spectrum was close to that of Pd_13_ clusters (CN = 5.5) generated in Pd/H-Y through reduction with 8% H_2_, which were active in the Heck reaction between bromobenzene and styrene [8]. Although it was difficult to confirm that the observed Pd cluster was the active species, taking into account that leached Pd in equilibrium with metal Pd has been reported to be the active species in many literature reports [17,18], the Pd clusters might be the precursor for the evolution of catalytic activity.

### 2.4. Effect of H_2_ Bubbling on the Catalytic Reactions of Pd/USY

**Figure 4:**
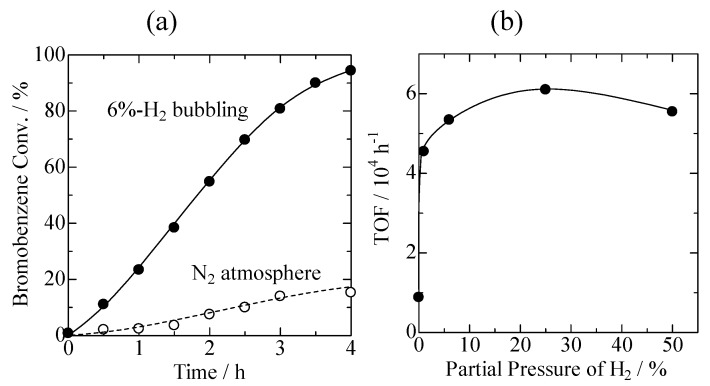
(a) Conversion of bromobenzene plotted as a function of the reaction time; (b) Turnover frequencies plotted as a function of partial pressure of H_2 _in the Heck reaction between bromobenzene and styrene over 0.4 wt %-Pd/USY. USY was prepared by steaming of NH_4_-Y at 873 K.

Heck coupling reactions were performed over Pd loaded on USY prepared by steam treatment of NH_4_-Y zeolite at 873 K. In order to activate the Pd/USY catalyst, a 6%-H_2_ flow at a rate of 30 mL min^−1^ was fed into the reactant solution during the catalytic reactions using a glass capillary. It should be emphasized that a small amount of 0.4 wt % Pd/USY (5 mg) was used with respect to 30 mmol of bromobenzene, corresponding to 0.00063 mol % of Pd. We found that Pd/USY worked very efficiently in Heck reactions when H_2_ bubbling was applied during reactions. Figure 4(a) shows a typical change in the conversion of bromobenzene with time of the reaction with styrene. Without the H_2_-bubbling treatment, the activity of Pd/USY was low, with a conversion of 15% being obtained at 4 h. In marked contrast to this, the conversion of bromobenzene reached 94% in 4 h when 6%-H_2_ bubbling was used, and the Pd TON reached 150,000. The effect of the 6%-H_2_ bubbling on the Heck reaction is therefore significant. Figure 4(b) shows the TOF plotted as a function of the partial pressure of H_2_. The addition of only 1% H_2_ to Ar was effective at enhancing the catalytic activity of Pd/USY significantly. TOF were slightly depended on the H_2_ partial pressure; the highest catalytic activity was attained at an H_2_ pressure of 25%.

### 2.5. Effect of the Thermal Treatment Temperature of the USY Support

**Figure 5:**
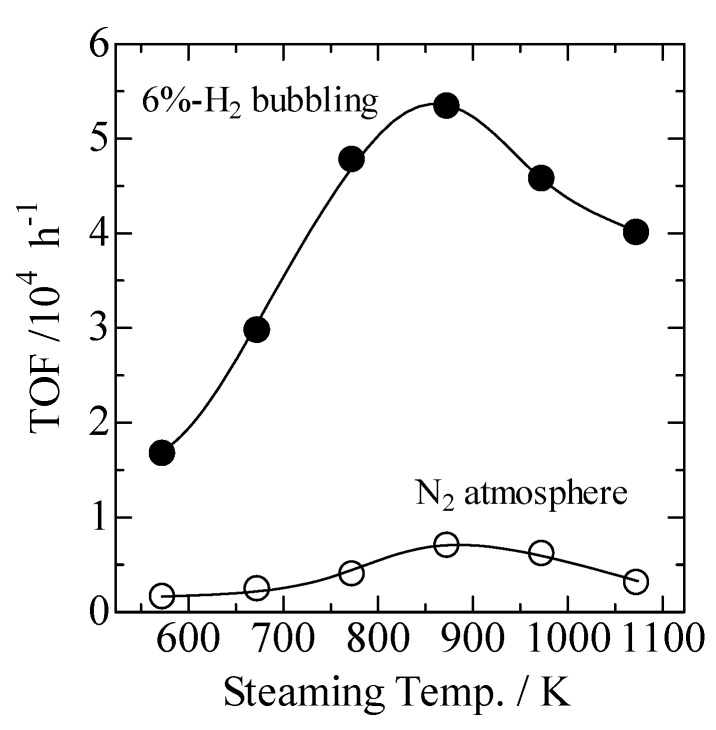
Turnover frequencies plotted as a function of the steaming temperature in the reaction between bromobenzene and styrene. Catalyst, Pd/USY. 6%-H_2_ bubbling was applied during reactions.

Figure 5 shows the turnover frequency (TOF) plotted as a function of the steam-treatment temperature for the preparation of USY. TOFs were calculated based on the conversion of bromo-benzene at 2 h from the beginning of the reaction and the amount of Pd present in Pd/USY measured by inductive coupled plasma (ICP) analysis. The TOF increased with increasing steam-treatment temperature, with the highest activity being reached at 873 K. The activity declined on further increasing the steaming temperature. These data indicated the remarkable effect of steam-treatment conditions on the catalytic performance of Pd. The maximum TOF obtained in Pd loaded on USY prepared at 873 K was 5.3×10^4^ h^−1^. The value was higher than those of previous reports such as Pd/MgO (TOF = 70 h^−1^ [19]), Pd/SiO_2_ (TOF = 110 h^−1^ [20]) and ferrocenylamine-derived palladacycles (TOF = 140 h^−1^ [21]). The optimum condition for Heck reaction (873 K, 100% H_2_O) was much severer than that for Suzuki reactions where USY was prepared by streaming with 18% H_2_O at 773 K [15]. Nevertheless, we found that that the optimum temperature for preparation of USY zeolite agreed with that of the maximum amount of OH_strong_ was obtained as measured by acid characterizations. The fact suggested that the strong Brønsted acid sites generated as the result of steaming of NH_4_-Y and subsequent treatment with ammonium nitrate solution acted as the anchor to keep the dispersed form during reactions [22].

### 2.6. Effect of Supports, H_2_ Bubbling and Solvents

Heck coupling reaction between bromobenzene and styrene was carried out over Pd/USY (steaming temperature: 873 K) in various kinds of solvents. The TONs reached at 20 h are compared in Table 3. A high TON of 630,000 was obtained in DMAc. TONs obtained in dimethylformamide (DMF) and *N*-methylpyroridone (NMP) were 390,000 and 100,000, respectively, which were lower than that obtained in DMAc. The reaction did not proceed in *o*-xylene as a solvent. The results point to the importance of choosing DMAc as the solvent.

**Table 3:** Heck reactions between bromobenzene and styrene catalyzed by Pd/USY in various kinds of solvents.^a^

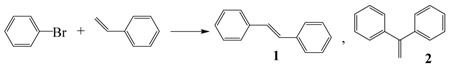						
Entry	Condition	Conv.^b^ / %	Yield of 1 / %	Yield of 2 / %	Material balance	TON
1	DMAc	98	96	1.4	99	630,000
2	NMP	80	62	0.9	82	390,000
3	DMF	18	16	0.2	99	100,000
4	*o*-xylene	0	0	0	100	0

^a^ Reaction conditions: Ph-Br (120 mmol), styrene (180 mmol), catalyst (5 mg), CH_3_COONa (144 mmol), solvent (120 mL), 413 K, 20 h. 6%-H_2_ bubbling (30 mL min^−1^) was applied during reactions; ^b^ Conversion of bromobenzene.

The TONs of Pd loaded on different types of supports are compared in Table 4. DMAc was employed as a solvent. Pd/USY (steaming temperature: 873 K) activated with 6%-H_2_ bubbling exhibited the highest activity (entry 3, TON = 630,000). The H_2_ bubbling was effective in enhancing the catalytic activity not only in Pd/USY, but also in Pd loaded on Al_2_O_3_ (entry 7, 8) or activated carbon (entry 9, 10). In a similar way, the TON of Pd(OAc)_2_ dissolved in DMAc increased from 170,000 (entry 11, without H_2_ bubbling) to 420,000 (entry 12) with H_2_ bubbling, indicating that H_2_ bubbling was also effective in a homogeneous catalyst. Hydrogenated or dehalogenated products were hardly observed in every reaction. It has been reported that the hydrogenation activity decreased as the size of the Pd cluster decreased [23]. Therefore it is assumed that the hydrogenation of styrene or products was suppressed by the formation of Pd clusters on USY zeolites. The reaction was also performed with Pd/USY under an atmosphere of 6% H_2_ for a comparison. That is to say, a 6%-H_2_ flow was introduced at the upper end of the flask to keep the atmosphere in the flask at 6% H_2_. The reaction over the 6%-H_2_ atmosphere was also effective at enhancing the activity of the Pd/USY (entry 2, TON = 600,000). In order to obtain insight into the role of hydrogen, hydrogen bubbling was stopped when the temperature of the solution reached 413 K. The obtained TON after 20 h from the begging of the reaction was 440,000 (entry 4), which was lower than that obtained by continuous hydrogen bubbling (entry 3, TON = 630,000). The observation was similar to that of Suzuki reaction. The fact suggested that the dissolved hydrogen had significant effect not only in the formation of active Pd species but also in the mechanism of Heck reaction. The role of hydrogen was not clearly understood at this stage, one hypothesis was that the dissolved hydrogen promoted the reductive elimination of products through the formation of Pd-H adducts [24].

**Table 4:** Heck reactions between bromobenzene and styrene catalyzed by Pd supported on different kinds of supports.^a^

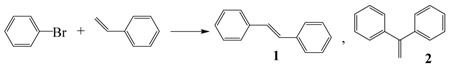							
Entry	Catalyst	Condition	Conv.^b^/ %^b^	Yield of 1 / %	Yield of 2 / %	Material balance	TON
1	Pd/USY	N_2_ atmosphere	47	37	0.5	90	300,000
2	Pd/USY	6%-H_2_ atmosphere	96	84	1.1	89	600,000
3	Pd/USY	6%-H_2_ bubbling	98	96	1.4	99	630,000
4	Pd/USY	6%-H_2_ bubb., N_2_^c^	70	60	0.8	92	440,000
5	Pd/H-Y	N_2_ atmosphere	9	6	0.1	98	55,000
6	Pd/H-Y	6%-H_2_ bubbling	11	10	0.2	99	70,000
7	Pd/Al_2_O_3_	N_2_ atmosphere	52	46	0.6	95	330,000
8	Pd/Al_2_O_3_	6%-H_2_ bubbling	71	61	0.8	90	460,000
9	Pd/AC	N_2_ atmosphere	17	13	0.1	96	110,000
10	Pd/AC	6%-H_2_ bubbling	54	44	0.6	90	430,000
11	Pd(OAc)_2_	N_2_ atmosphere	27	20	0.2	93	170,000
12	Pd(OAc)_2_	6%-H_2_ bubbling	67	58	0.7	92	420,000

^a^ Conditions: See the caption of Table 3; ^b^ Conversion of bromobenzene; ^c^ 6%-H_2_ bubbling was stopped when the temperature reached 413 K.

### 2.7. Heck Reactions Using Various Kinds of Substrates

Table 5 gives the results of reactions carried out in the presence of Pd/USY using various of bromobenzene, -pyridine, -thiophene, -quinoline, -naphthalene derivatives under 6%-H_2_ bubbling. The USY support was prepared by steaming of NH_4_-Y at 873 K with 100% H_2_O. High TONs of up to 630,000 were obtained with various bromobenzene and bromonaphthalene derivatives, where the cross-coupling reaction proceeded almost quantitatively, except for several substrates. We found that the Pd/USY was also applicable to the heteroaromatic compounds with TON = 1,400–5,900 (entry 13–16). We tried to repeatedly use the Pd/USY in Heck reactions after separating the catalyst from reaction mixture by filtration. However, the use of the recycled Pd/USY was difficult; probably due to the aggregation of Pd. Despite this, it is notable that the Pd/USY is useful in Heck reactions considering that high TOF and TON values were obtained with different substrates.

**Table 5:** Heck reactions between bromobenzene derivatives and styrene catalyzed by Pd/USY.^a^

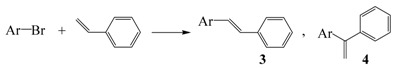								
Entry	Ar-Br	Pd / mol%	Time / h	Conv. / %	Yield of 3 / %	Yield of 4 / %	Material balance	TON
1	Bromobenzene	1.6×10^−4^	20	98	96	1.4	99	630,000
2	4-Bromoacetophenone	2.3×10^−4^	24	96	91	0.8	96	410,000
3	3-Bromoacetophenone	2.3×10^−4^	24	98	85	0.8	88	420,000
4	4-Bromobenzaldehyde	3.7×10^−4^	27	91	88	0.8	98	240,000
5	4-Bromotoluene	1.9×10^−4^	24	99	97	1.5	100	520,000
6	3-Bromotoluene	2.3×10^−4^	20	42	34	0.6	92	190,000
7	2-Bromotoluene	4.6×10^−4^	20	99	79	0.8	81	220,000
8	4-bromobenzonitrile	4.7×10^−4^	20	80	76	1.8	97	170,000
9	4-bromoanisole	4.7×10^−4^	20	57	56	0.6	99	140,000
10	4-Bromonitrobenzene	4.7×10^−4^	20	55	53	0.6	99	120,000
11	1-Bromonaphthalene	1.6×10^−4^	20	93	88	1.1	97	590,000
12	2-Bromonaphthalene	1.6×10^−4^	20	87	84	1.2	99	550,000
13	3-Bromothiophene	1.6×10^−^^2^	2	80	73	1.3	95	5,900
14	2-Bromothiophene	1.6×10^−^^2^	2	53	26	0.4	73	2,100
15	3-Bromopyridine	2.5×10^−^^2^	20	75	61	0.8	86	2,400
16	1-Bromoquinoline	6.2×10^−^^2^	1.5	99	87	1.4	88	1,400

^a^ Conditions: See the caption of Table 3. DMAc was used as solvent. The scales of all the reagents were changed, while the catalyst weight was fixed at 5 mg. Reaction was carried out under 6%-H_2_ bubbling (30 mL min^−1^). The USY zeolite was prepared by steaming of NH_4_-Y at 873 K with 100% H_2_O.

Table 6 gives the results of reactions using derivatives of bromobenzene and *tert*-butyl acrylate. Similarly to the case of the reactions using styrene, high TONs of up to 570,000 were obtained with various substrates.

**Table 6:** Heck reactions between bromobenzene derivatives and *tert*-butyl acrylate catalyzed by Pd/USY.^a^

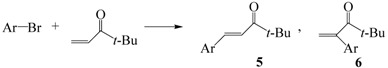								
Entry	Ar-Br	Pd /mol%	Time / h	Conv./%	Yield of 5 / %	Yield of 6 / %	Material balance	TON
1^b^	4-Bromoacetophenone	1.9×10^−4^	24	76	66	0.1	94	410,000
2^b^	4-Bromobenzaldehyde	1.6×10^−4^	24	91	87	0.1	98	570,000
3^b^	4-Bromonitrobenzene	1.6×10^−4^	30	88	82	0.1	95	530,000
4^b^	4-Bromobenzonitrile	1.6×10^−4^	24	98	92	0.1	95	570,000
5^c^	1-Bromonaphthalene	6.2×10^−^^3^	3.5	93	87	0.2	82	15,000

^a^ The USY zeolite was prepared by steaming of NH_4_-Y at 873 K with 100% H_2_O; ^b^ Reaction conditions: Ar-Br (120 mmol), *tert*-butyl acrylate (180 mmol), catalyst (5 mg), CH_3_COONa (144 mmol), DMAc (120 mL), 413 K; ^c^ Reaction condition: 1-Bromonaphthalene (3 mmol), *tert*-butyl acrylate (4.5 mmol), catalyst (5 mg), CH_3_COONa (3.6 mmol), DMAc (3 mL), 413 K.

### 2.8. Reactions Using 4-Chloroacetophenone

Finally, Heck coupling reactions between chlorobenzene derivatives and styrene was carried out over Pd/USY in *N*-methylpyrrolidone (NMP) as a solvent (Table 7).

**Table 7:** Heck reactions between chlorobenzene derivatives and styrene catalyzed by Pd/USY.^a^

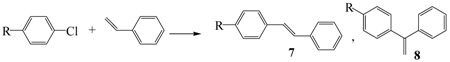							
Entry	Ar-Cl	Condition	Conv. of Ar-Cl /%^c^	Yield of 7 / %	Yield of 8 / %	Material balance ^d^	TON
1^b^	4-chloroacetophenone	N_2_ atmosphere	44	33	0.3	90	17,000
2^b^	4-chloroacetophenone	6%-H_2_ bubbling	98	97	0.7	100	49,000
3	4-chloroacetophenone	6%-H_2_ bubbling	0.5	0.5	0	100	680
4^b^	4-chlorobenzonitrile	6%-H_2_ bubbling	65	48	0.4	83	35,000
5^b^	4-chloronitrobenzene	6%-H_2_ bubbling	-	10	0.1	-	2,600
6^b^	chlorobenzene	6%-H_2_ bubbling	-	2.1	0	-	580
7^b^	4-chloroanisole	6%-H_2_ bubbling	-	0.4	0	-	100

^a^ Reaction conditions: chlorobenzene derivatives (10 mmol), styrene (15 mmol), catalyst (5 mg), CH_3_COONa (12 mmol), NMP (10 mL), 413 K, 20 h. The USY zeolite was prepared by steaming of NH_4_-Y at 873 K with 100% H_2_O; ^b^ TBAB (1 mmol) was added; ^c,d^ Conversion and yield of the reactions 5–7 were not displayed because much portion of the substrates (Ar-Cl) were evaporated during reactions.

The reactions were carried out with or without addition of *tert*-tetrabutylammonium bromide (TBAB). Without the addition of TBAB, the activity was almost negligible (entry 3). However, significant improvement in activity was achieved by the addition of 1 mmol of TBAB in the reaction using 4-chloroacetophenone as the substrate (entry 2) as reported earlier [25,26]. The positive effect of H_2_ bubbling was confirmed again when the comparison was made between the reactions of entry 1 (TON = 17,000) and entry 2 (TON = 49,000). Although high conversions and TONs were obtained with the use of electron-withdrawing substituents such as 4-chloroacetophenone and -benzonitrile (entry 2, 4), the activity became lower when chlorobenzene and 4-chloroanisole were used for reactions (entry 6, 7).

## 3. Conclusions

We have found that the Heck coupling activity of Pd/USY as well as that of Pd loaded on other supports was enhanced by the application of H_2_ bubbling during reaction. Moreover, preparation conditions of the USY support influenced the catalytic performance of Pd significantly. High TON values-up to 630,000-were obtained after optimization of the steam-treatment conditions. The Pd/USY catalysts were applicable in various Heck reactions, including those using bromobenzenes, -naphthalenes, -heteroaromatic and chlorobenzene derivatives. Characterization of the acid properties of the USY zeolites revealed that the OH_strong_ group generated as a result of steam treatment was important in evolution of high catalytic activity in Pd/USY.

## 4. Experimental

### 4.1. Preparation of USY Zeolites

Na-Y zeolite (320NAA) supplied by the Tosoh Corp. (Tokyo, Japan) was employed as the starting material for the preparation of USYs. The Na-Y was ion-exchanged three times with a solution of NH_4_NO_3_ (0.5 mol L^−1^) at 353 K to give NH_4_-Y. USY was prepared from NH_4_-Y zeolites by treatment with H_2_O vapor diluted with an N_2_ flow. Concentration of H_2_O vapor was 100 vol%. NH_4_-Y (5 g) was placed in a quartz tube and treated with H_2_O vapor for 1 h at 573–1073 K. The flow rate was 50 mL min^−1^. The obtained USY was ion-exchanged three times with NH_4_NO_3_ (0.5 mol L^−1^) at 353 K to give NH_4_-USY. The completion of ion exchange of H^+^ with NH_4_^+^ was confirmed by TPD of NH_3_, showing that 93% of H^+^ had been replaced with NH_4_^+^. The NH_4_-USY was then heated to 573 K in an N_2_ stream to partially remove NH_3_.

### 4.2. Loading of Pd on USY Zeolites

Pd was then introduced to the calcined USY by an ion-exchange method using Pd(NH_3_)_4_Cl_2_ solution (3.8 × 10^−4^ mol L^–1^; Aldrich, St. Louis, MO, USA) at room temperature (r.t.). That is to say, USY zeolite (4 g) was added to an aqueous solution of Pd(NH_4_)Cl_2_ (400 mL). The suspension was stirred for 4 h, followed by washing with deionized H_2_O. The obtained solid was dried overnight in an oven at 323 K. Pd was loaded on H-Y with an ion-exchange method using Pd(NH_4_)Cl_2_, in a similar way for the preparation of Pd/USY. The H-Y was prepared through calcination of NH_4_-Y at 773 K. The Pd loading of all samples was 0.4 wt %, as measured by ICP analysis. Pd (0.4 wt %) loaded on Al_2_O_3_ (JRC-ALO-3, Catalysis Society of Japan) and activated carbon (Wako Chemicals Ltd., Osaka, Japan) were prepared by the impregnation method using a Pd(NH_3_)_4_Cl_2_ solution. 

### 4.3. Catalytic Reactions

Under typical conditions, bromobenzene (120 mmol; Tokyo Kasei Chemicals Ltd., Japan), styrene (180 mmol; Tokyo Kasei Chemicals Ltd., Japan), CH_3_COONa (144 mmol; Wako Chemicals Ltd., Osaka, Japan), DMAc (solvent, 120 mL, Wako Chemicals Ltd., Osaka, Japan), and 0.4 wt% Pd/USY catalyst (5 mg, Pd: 1.9 × 10^−^^7^ mol) were used for the Heck reactions. A 6% H_2_/94% Ar flow at a rate of 30 mL min^−1^ was introduced into the reactant solution using a glass capillary tube during reactions. A three-necked flask (300 mL) was placed in an oil bath, preheated to the required temperature, and subjected to vigorous stirring. The reaction was performed at 413 K. Heck reactions using chlorobenzene derivatives were performed under similar conditions, except for the use of NMP as the solvent. After reaction, the reaction mixture was cooled to r.t. and the solution was analyzed using a Shimadzu 2010 Gas Chromatograph equipped with an InertCap 5 (30 m) capillary column (Shimadzu Corp., Kyoto, Japan). Tridecane was used as the internal standard.

### 4.4. Characterization of the Acid Properties of USY Zeolites

A Fourier-transform IR (FT-IR) spectrometer (Perkin-Elmer Spectrum-One; Perkin-Elmer, Waltham, MA, USA) and a mass spectrometer (Pfeiffer QME200; Pfeiffer, Asslar, Germany) were connected with a vacuum line kept at 3.3 kPa through which He was allowed to flow as the carrier (flow rate: 110 mL min^−1^). An IR beam was transmitted to a self-compressed disk (5 mg, and 10 mm in diameter). After evacuation of the sample at 773 K, IR spectra were recorded before NH_3_ adsorption at 10 K intervals from 373 to 773 K, while the temperature was increased at a rate of 10 K min^−1^ (*N*(T), recorded). The bed temperature was then lowered to 373 K, at which point NH_3_ was adsorbed at 13 kPa, and then gas-phase NH_3_ was evacuated for 30 min. IR spectra were again measured at 10 K intervals from 373 to 773 K, while the temperature was raised (*A(T)*, recorded). The difference spectrum, *i.e.*, *A*(*T*) − *N*(*T*), was calculated at each temperature, and changes in IR absorptions were observed to identify the absorptions of NH_4_^+^ and NH_3_ adsorbed on the surface. Changes in OH bands were also detected in the difference spectra as negative peaks. The difference spectra with respect to temperature, *i.e.*, −d(*A*(*T*) − *N*(*T*))/d*T*, were calculated at selected band positions. In the present study, the area of IR absorption was quantified in the absorption range. The IR-TPD was compared with the MS-measured TPD of NH_3_ (m/e, 16) to identify the nature of the adsorption site for the desorbed NH_3_. Detailed data analysis was described elsewhere [15].

### 4.5. Pd K-edge EXAFS Measurements

Pd K-edge EXAFS data were obtained from synchrotron radiation experiments performed at the BL01B1 station, with the approval of the Japan Synchrotron Radiation Research Institute (JASRI) (Proposal No. 2010A1072). Pd K-edge XAFS data were collected in the quick mode; a Si(111) monochromator was continuously moved from 4.75° to 4.40° for 5 min. The beam size was 5 mm (horizontal) × 0.8 mm (vertical) at the sample position. For the Pd K-edge XAFS measurements, 6% H_2 _was bubbled through a mixture of Pd/USY in DMAc, and the treated Pd/USY was transferred to a plastic cell at room temperature without contact with air. The X-ray path length of the plastic cell was 2 cm.

## References

[1] Beletskaya I.P., Cheprakov A.V. (2000). The heck reaction as a sharpening stone of palladium catalysis. Chem. Rev..

[2] Beletskaya I.P., Cheprakov A.V. (2004). Palladacycles in catalysis—A critical survey. J. Organomet. Chem..

[3] Dupont J., Consorti C.S., Spencer J. (2005). The potential of palladacycles: More than just precatalysts. Chem. Rev..

[4] Peris E., Crabtree R.H. (2004). Recent homogeneous catalytic applications of chelate and pincer *N*-heterocyclic carbenes. Coord. Chem. Rev..

[5] Mehnert C.P., Weaver D.W., Ying J.Y. (1998). Heterogeneous Heck catalysis with palladium-grafted molecular sieves. J. Am. Chem. Soc..

[6] Heidenreich R.G., Kohler K., Krauter J.G.E., Pietsch J. (2002). Pd/C as a highly active catalyst for Heck, Suzuki and Sonogashira reactions. Synlett.

[7] Demel J., Cejka J., Stepnicka P. (2007). The use of palladium nanoparticles supported on MCM-41 mesoporous molecular sieves in Heck reaction: A comparison of basic and neutral supports. J. Mol. Catal. A.

[8] Okumura K., Nota K., Yoshida K., Niwa M. (2005). Catalytic performance and elution of Pd in the Heck reaction over zeolite-supported Pd cluster catalyst. J. Catal..

[9] Dams M., Drijkoningen L., Pauwels B., Van Tendeloo G., De Vos D.E., Jacobs P.A. (2002). Pd-zeolites as heterogeneous catalysts in Heck chemistry. J. Catal..

[10] Djakovitch L., Koehler K. (2001). Heck reaction catalyzed by Pd-modified zeolites. J. Am. Chem. Soc..

[11] Chandrasekhar S., Narsihmulu C., Sultana S.S., Reddy N.R. (2002). Poly(ethylene glycol) (PEG) as a reusable solvent medium for organic synthesis. Application in the Heck reaction. Org. Lett..

[12] Ribiere P., Declerck V., Nedellec Y., Yadav-Bhatnagar N., Martinez J., Lamaty F. (2006). Synthesis of novel poly(ethylene glycol) supported benzazepines: the crucial role of PEG on the selectivity of an intramolecular Heck reaction. Tetrahedron.

[13] Okumura K., Matsui H., Tomiyama T., Sanada T., Honma T., Hirayama S., Niwa M. (2009). Highly dispersed Pd species active in the Suzuki-Miyaura reaction. Chem. Phys. Chem..

[14] Okumura K., Matsui H., Sanada T., Arao M., Honma T., Hirayama S., Niwa M. (2009). Generation of the active Pd cluster catalyst in the Suzuki-Miyaura reactions: Effect of the activation with H_2_ studied by means of quick XAFS. J. Catal..

[15] Okumura K., Tomiyama T., Okuda S., Yoshida H., Niwa M. (2010). Origin of the excellent catalytic activity of Pd loaded on ultra-stable Y zeolites in Suzuki-Miyaura reactions. J. Catal..

[16] Beyerlein R.A., ChoiFeng C., Hall J.B., Huggins B.J., Ray G.J. (1997). Effect of steaming on the defect structure and acid catalysis of protonated zeolites. Top. Catal..

[17] Prockl S.S., Kleist W., Gruber M.A., Kohler K. (2004). *In situ* generation of highly active dissolved palladium species from solid catalysts - A concept for the activation of aryl chlorides in the Heck reaction. Angew. Chem.-Int. Ed..

[18] Reetz M.T., de Vries J.G. (2004). Ligand-free Heck reactions using low Pd-loading. Chem. Commun..

[19] Chen F.X., Toh K., Shen S.C., Gan G.J. (2007). Porous magnesia as solid base for ligand-free Heck reaction. Catal. Commun..

[20] Huang L., Wang Z., Ang T.P., Tan J., Wong P.K. (2006). A novel SiO_2_ supported Pd metal catalyst for the Heck reaction. Catal. Lett..

[21] Wang H.X., Wu H.F., Yang X.L., Ma N., Wan L. (2007). Highly active ferrocenylamine-derived palladacycles for carbon-carbon cross-coupling reactions. Polyhedron.

[22] Tomiyama T., Okumura K., Niwa M. (2011). Enhancement in cracking activity of USY zeolites treated with ammonium nitrate solution. Chem. Lett..

[23] Wilson O.M., Knecht M.R., Garcia-Martinez J.C., Crooks R.M. (2006). Effect of Pd nanoparticle size on the catalytic hydrogenation of allyl alcohol. J. Am. Chem. Soc..

[24] Xu L.Q., Zhang Z.C., Sachtler W.M.H. (1992). Effect of zeolite protons on Pd particle-size and H_2_ chemisorption. J. Chem. Soc.-Faraday Trans..

[25] Botella L., Najera C. (2004). Synthesis of methylated resveratrol and analogues by Heck reactions in organic and aqueous solvents. Tetrahedron.

[26] Kleist W., Lee J.K., Kohler K. (2009). Pd/MO_x_ materials synthesized by sol-gel coprecipitation as catalysts for carbon-carbon coupling reactions of aryl bromides and chlorides. Eur. J. Inorg. Chem..

